# Building Teacher Capacity for Inclusive Practice With Autistic Pupils: A Mixed-Methods Study of Self-Efficacy, Training, Attitudes, and School Context

**DOI:** 10.1177/13623613261478886

**Published:** 2026-09-03

**Authors:** Anna Cook, Alice Boddy

**Affiliations:** 1 University of Surrey, UK

**Keywords:** autism, inclusive education, teacher self-efficacy, training, attitudes, secondary school

## Abstract

Autistic pupils in mainstream secondary schools often experience unmet needs, yet limited research has examined the combined influence of multiple theoretically relevant predictors of teachers’ self-efficacy and intentions to implement inclusive practice for autistic pupils. This mixed-methods study surveyed 126 staff from six secondary schools, assessing autism-specific training, perceived inclusiveness of the school environment, experience with autistic pupils, neurodiversity-affirming attitudes, personal connection to autism, and staff role. Teacher self-efficacy and intentions for inclusive practice were measured using adapted scales, and hierarchical multiple regression identified significant predictors. Autism-specific training, perceived quality of the inclusive environment, years of experience with autistic pupils, classroom teacher role, and neurodiversity-affirming attitudes predicted higher self-efficacy. Together, these explained 42.1% of the variance. Intentions for inclusive practice were predicted by neurodiversity-affirming attitudes and training, explaining 27.5% of the variance. Thematic analysis of open-text responses revealed practical adjustments including clear communication, sensory adaptations, and emotional regulation supports, alongside challenges such as resource constraints, complex classroom dynamics, and inconsistent institutional support. Findings highlight the value of targeted autism-specific training and fostering neurodiversity-affirming attitudes, supported by inclusive school environments, to strengthen teacher capacity and sustain inclusive practice.

**Lay Abstract:**

Many autistic pupils in mainstream secondary schools do not have their needs fully met. This can affect their learning, well-being, and opportunities to take part in school life. Teachers play a vital role in making schools more inclusive, but their ability and motivation to do so can be influenced by many different factors. Most research has looked at these factors separately. This study is one of the first to examine several important influences together, to see which are most strongly linked to teachers feeling confident (self-efficacy) and intending to use inclusive practices for autistic pupils. We worked with 126 staff from six secondary schools, including teachers, leaders, teaching assistants, and other support staff. We looked at the impact of autism-specific training, school environment, experience with autistic pupils, attitudes towards neurodiversity, personal connection to autism, and staff role. Participants were also asked to describe examples of strategies they use to support autistic pupils, and the challenges they face. We found that confidence was highest among staff who had autism-specific training, worked in a more inclusive school environment, had more years of experience with autistic pupils, were classroom teachers (rather than in leadership roles), and had more positive, neurodiversity-affirming attitudes. Together, these factors explained more than two fifths (about 42%) of the differences in how confident staff felt. Intentions to use inclusive practices were strongest among staff with neurodiversity-affirming attitudes and autism-specific training, and these two factors together explained just more than a quarter (about 28%) of the differences in how strong those intentions were. Staff described a wide range of helpful strategies, such as giving clear instructions, adapting sensory environments, and supporting emotional regulation. However, they also reported challenges such as limited time, resources, and inconsistent support from school systems. The findings show that targeted autism-specific training, positive attitudes towards neurodiversity, and inclusive school environments can work together to strengthen teacher confidence and commitment to inclusion. Removing systemic barriers will help ensure that staff can put these skills and values into practice to benefit autistic pupils.

## Introduction

The needs of autistic pupils are frequently reported as unmet in U.K. secondary schools (Goodall, 2018, 2019; Humphrey & Lewis, 2008; National Autistic Society [NAS], 2021). Studies have identified multiple factors that contribute to these inequities, including teachers’ preparedness to teach autistic pupils, the inclusivity of the school environment, teacher self-efficacy, and attitudes towards inclusion. However, most studies examine these factors in isolation or in limited combinations, leaving unanswered questions regarding their interrelationships and the most influential predictors of positive outcomes.

### Teacher Preparedness for Inclusion of Autistic Pupils

Inclusion in education refers to the process of ensuring that all pupils, regardless of disability or difference, participate fully in the academic and social life of the school, with equitable access to learning opportunities, resources, and support (United Nations Educational, Scientific and Cultural Organization [UNESCO], 2017). Globally, many countries have expanded the inclusion of pupils with special educational needs and disabilities (SEND) within mainstream settings. However, inclusion can present significant challenges for teachers (Florian & Becirevic, 2011; McKay, 2016). Structural barriers include resource constraints and insufficient teacher preparation (Hamilton & Cook, 2026). In the current English system, initial teacher education programmes are required to prepare trainees to work with pupils with special educational needs, and autism is referenced within national guidance, but there is no nationally mandated stand-alone module on autism or neurodivergent learners. Coverage is therefore variable, and evidence from a large survey for the NAS (2023) indicated that most teachers in England had no more than half a day of autism-specific training, suggesting that both initial teacher education and subsequent continuing professional development (CPD) provide highly variable and often very limited coverage of autism.

Despite policy commitments to inclusion, research reveals wide variation in teacher preparedness to educate pupils with SEND (Florian et al., 2010; Florian & Pantic, 2017; Mintz et al., 2020). Schools often lack the funding and specialist staff needed to fully support autistic pupils (Avramidis et al., 2000; Iadarola et al., 2014). While many teachers endorse inclusion in principle, substantial gaps persist in nuanced understanding of autism (Sharma et al., 2008).

The majority of teachers report feeling underprepared to teach autistic pupils (Anglim et al., 2018; Lindsay et al., 2013; Loiacono & Allen, 2008; Loiacono & Valenti, 2010; Symes & Humphrey, 2011). This is particularly significant given evidence that targeted, autism-specific professional development can improve teacher outcomes (Alassaf, 2025; Love et al., 2019; Nolan & Hannah, 2019; Ryan & Mathews, 2022). In contrast, one-off or generic training often leaves teachers unprepared, a concern reflected in U.K. data indicating high demand for autism-focused training (Hamilton & Cook, 2026; NAS, 2023). Inadequate preparation has profound consequences for autistic pupils, who may struggle both academically and socially without informed and adaptive teaching approaches (Goodall, 2018; Goodall & MacKenzie, 2019; Neal & Frederickson, 2016; Poon et al., 2014; Saggers, 2015; Saggers et al., 2011).

Recent international studies further demonstrate that educators who have undertaken autism-specific training exhibit greater autism knowledge and more positive inclusion attitudes than those without such training (Alassaf, 2025). Similarly, multiple studies show that teacher training enhances self-efficacy for inclusive education (Boyle et al., 2013; Forlin et al., 2014).

Related to teachers’ preparedness is the concept of teacher self-efficacy. This refers to a teacher’s belief in their capacity to successfully execute teaching tasks and facilitate desired student outcomes (Tschannen-Moran & Woolfolk Hoy, 2001). Research over the past two decades has consistently linked higher teacher self-efficacy to positive professional outcomes, including job satisfaction (Malinen & Savolainen, 2016; Vieluf et al., 2013), constructive engagement with students (Dembo & Gibson, 1985; Woolfolk & Hoy, 1990), effective classroom management (Ryan et al., 2015), greater teaching enthusiasm (Allinder, 1994), and more inclusive practices (Sharma & Jacobs, 2016; Sharma & Sokal, 2016).

### Teacher Attitudes Towards Inclusion and Neurodiversity

Numerous studies have examined teacher attitudes towards inclusive education, identifying both knowledge of disabilities and experience in inclusive settings as key influences (Alassaf, 2025; Nolan & Hannah, 2019; Park & Chitiyo, 2011; Van Mieghem et al., 2018). Professional development has a demonstrably positive effect on attitudes, particularly when it addresses areas of low confidence and includes mastery experiences (Avramidis & Norwich, 2002; Beh-Pajooh, 1992; Forlin et al., 2014; Lai et al., 2016; Leyser et al., 2011; Sharma et al., 2008; Shimman, 1990).

Positive correlations between attitudes and self-efficacy have been documented in multiple studies, including meta-analytic (Yada et al., 2022) and systematic review evidence (Wray et al., 2022), although the causal direction remains unclear (Savolainen et al., 2022). Together, self-efficacy and positive attitudes are strong predictors of inclusive practice (Alnahdi & Schwab, 2021; Boyle & Anderson, 2020; Hellmich et al., 2019; Savolainen et al., 2022; Yada et al., 2018).

Teachers’ beliefs have an impact on their inclusive practices, for example, teachers who attribute pupils’ problems to an interaction between the pupil and the environment are more likely to adopt inclusive practices than those who assume that a disability is inherent in the individual pupil (Avramidis & Norwich, 2002; De Boer et al., 2011; Schwab, 2018; Savolainen et al., 2020). The neurodiversity paradigm reframes autism as another form of human diversity, as opposed to prior “medical” models which conceptualise autism in terms of deficits to be “treated” or overcome (Chapman, 2021; Fletcher-Watson, 2022; Walker, 2014). Neurodiversity-affirming attitudes therefore emphasise strengths, value difference, and advocate for systemic adaptations rather than conformity. Empirical studies on educators’ attitudes towards neurodiversity are scarce. However, it is reasonable to expect that endorsement of such values would positively influence inclusive practice.

### The Role of Practical Experience

Alongside formal training, direct classroom experience with autistic pupils can be pivotal in shaping teachers’ confidence, attitudes, and instructional strategies. Self-efficacy theory (Bandura, 1997) identifies mastery experiences – for example, successful engagement with real teaching challenges – as the most influential source of efficacy beliefs. In line with this, qualitative and survey research indicates that teachers who have worked with autistic pupils (or pupils with other special educational needs) often report increased confidence and more positive attitudes over time (Bešić et al., 2017; Hannah & Nolan, 2019). Regular, meaningful interaction appears to reduce anxiety, provide concrete strategies, and demonstrate the potential for diverse learners to thrive (Humphrey & Lewis, 2008; Huws & Jones, 2010; Yuker, 1994).

However, the relationship between experience and teacher self-efficacy is not uniformly supported. Some studies report no significant correlation between years of teaching experience and efficacy for supporting autistic students (Corona et al., 2017; Ruble et al., 2011). Conversely, Lo et al. (2014) found a positive correlation between teachers’ confidence and the extent of their experience with autistic pupils. Veteran teachers may develop more sophisticated adaptive skills (Le Roy & Simpson, 1996; Villa et al., 1996), suggesting that not only the quantity but also the quality and diversity of experiences are relevant to efficacy development.

### The Influence of the School Environment

The wider school ethos and environment also exert substantial influence over teachers’ capacity to implement inclusive practices. An inclusive school environment – characterised by supportive leadership, collaborative culture, adequate resources, and a shared commitment to diversity – can mitigate many barriers to inclusion and foster higher levels of teacher self-efficacy (Collie et al., 2012; Hoy & Woolfolk, 1993; Pas et al., 2012; Weisel & Dror, 2006).

Environmental supports extend beyond physical adaptations to encompass relational and cultural dimensions, such as trust, collegiality, and the consistent modelling of inclusive values (Cohen et al., 2009; Hoy & Woolfolk, 1993). Schools with adequate specialist staff, flexible learning spaces, and targeted supports are more likely to successfully include pupils with additional needs (Avramidis et al., 2000). Conversely, high pupil–teacher ratios, insufficient training opportunities, and inflexible curricula can significantly impede inclusion (Cook, 2024). For autistic pupils, environmental features such as low-sensory spaces, visual supports, and quiet areas can be particularly beneficial (Boddy & Cook, 2026), though these require deliberate investment and prioritisation.

### Personal Connections, Staff Roles, and Implications for Practice

There is some evidence that educators’ personal connections to neurodivergence – such as having an autistic family member or close friend – may enhance empathy, shape expectations, and increase willingness to make pedagogical adaptations (Austin, 2014; Robertson et al., 2003). While such connections may help to deepen commitment to inclusive values, it remains unclear whether they can substitute for targeted professional development.

Staff roles also affect the degree and nature of exposure to autistic pupils. Teaching assistants (TAs) and specialist support staff often engage in more sustained one-to-one interactions, potentially affording more mastery experiences and higher perceived competence in supporting autistic learners (Symes & Humphrey, 2012; Webster et al., 2013). Classroom teachers and school leaders, in contrast, may exert greater influence on structural and cultural aspects of inclusion but have less opportunity for intensive individualised interaction.

### Limitations of Existing Research

Prior research – outlined above – has identified multiple factors associated with teachers’ self-efficacy and inclusive practice for autistic pupils. However, most studies have examined these predictors in isolation or within narrow combinations. While some have explored multiple factors (e.g., Alassaf, 2025; Hosford & O’Sullivan, 2016; Humphrey & Symes, 2013; Love et al., 2019; Nolan & Hannah, 2019; Park & Chitiyo, 2011; Ryan & Mathews, 2022), few have incorporated all key predictors within a single, theoretically grounded model to evaluate their relative influence. Furthermore, much of the existing literature conflates generic SEND training with autism-specific training, despite important differences in content and relevance. Similarly, research on teacher attitudes frequently employs broad inclusion measures, omitting items that reflect neurodiversity-affirming perspectives or social justice orientations (Forlin et al., 2014; Hosford & O’Sullivan, 2016; Monsen et al., 2014; Sharma et al., 2012).

There is also limited quantification of the distinct impact of years of experience teaching autistic pupils, as opposed to general teaching experience. Overall, little is known about the combined influence of autism-specific training, inclusive climate, classroom experience, and neurodiversity-affirming attitudes on teacher self-efficacy and intentions to use inclusive practices.

### The Present Study

The present study addresses these gaps by examining multiple predictors of teachers’ self-efficacy for autism and intentions to use inclusive practices within a single analytical model. We surveyed 126 staff members – including classroom teachers, school leaders, TAs, and support staff – across six mainstream secondary schools in Southeast England. The study focused on secondary schools, a context where previous research suggests teacher attitudes towards inclusion may be less positive than in earlier educational phases. Compared with primary education, secondary schools are often larger and more departmentalised settings in which pupils are taught by many different teachers and move between classrooms throughout the day, making communication, coordinated planning, and relationship-building more difficult (Avramidis & Norwich, 2002; Rakap & Kaczmarek, 2010). These organisational features, together with more crowded, noisy, and academically pressured environments, may increase the sensory and social demands placed on autistic pupils and contribute to staff perceiving inclusion as more challenging in secondary contexts (Avramidis & Norwich, 2002; Nolan & Hannah, 2019; Rakap & Kaczmarek, 2010).

Based on prior literature, we hypothesised that (H1) teachers’ self-efficacy for autism and (H2) intentions to implement inclusive practices would be positively predicted by experiential factors (autism-specific training and greater direct experience with autistic pupils) and more proximal attitudinal and contextual factors (higher perceived quality of the inclusive school environment and neurodiversity-affirming attitudes). We also explored the potential influence of staff role and personal connection to autism, given the limited research on these variables.

Furthermore, the survey asked participants to describe examples of reasonable adjustments they used to support autistic pupils, to rate how likely they would be to use these adjustments, to identify perceived difficulties in providing support, and, where relevant, to comment on any changes to their practice following autism-specific training. Through this mixed-methods approach, the study seeks to advance understanding of how to strengthen educator capacity to meet the needs of autistic pupils, in line with current English policy goals for inclusive education, which emphasise building expertise in mainstream schools and improving access to high-quality, evidence-informed professional development for staff (Department for Education, *Every Child Achieving and Thriving*).

## Method

### Design

A cross-sectional correlational design was used to investigate predictors of teachers’ self-efficacy for autism and intentions to implement inclusive practices. (Although predictors are discussed in statistical terms, the cross-sectional design precludes causal inference or conclusions about temporal ordering.) In addition to quantitative measures, participants provided qualitative data on reasonable adjustments, their likelihood of use, challenges in supporting autistic pupils and changes in practice following training.

### Participants

G*Power analysis indicated that for a medium effect size (0.25), alpha = .05, and power = .80, with 10 predictor variables, the total sample size needed to be 118.

Participants were recruited from six suburban mainstream secondary schools in Southeast England, with varying socioeconomic profiles (free school meal eligibility ranged from 8.8% to 33%; national average = 24.6%). Five schools had specialist resource centres (SRCs) for autistic pupils, whereas one school had no SRC.

Of 126 total participants, 32 (25.4%) were male, 92 (73.0%) were female, one was non-binary and one was undisclosed. Ethnic composition: 117 (92.9%) White, three (2.4%) mixed/multiple ethnic groups, four (3.2%) Asian/Asian British, two (1.6%) undisclosed. The sample included 18 (14.3%) in leadership roles, 70 (55.6%) classroom teachers, 22 (17.5%) TAs, and 16 (12.7%) non-teaching support staff. 92 (73.0%) had a personal connection to autism (i.e., identified as autistic or had an autistic child/close family member/someone they knew well). Thirty-eight (30.2%) received autism-specific training from an external provider: special educational needs coordinator (SENCo) qualification, part of a higher education course (other than initial teacher training) or an autism-specific training course. Ninety-five (75.4%) learned about autism in their school CPD. Eighty-six (68.3%) had experience of working with autistic pupils for 5 or more years and 40 (31.7%) had fewer than 5 years’ experience.

The study received ethical approval from the University of Surrey Ethics Committee (Ref: FHMS 22-23 161 EGA).

### Procedure and Measures

Participants received the full study protocol and ethical information, including confirmation that their responses would remain anonymous, and they could withdraw at any time. Consent forms and surveys were administered online via *Qualtrics*. Average completion time was 18 min (range: 5–65 min). Demographic data collected included gender, ethnic background, highest qualification/level of education, and number of years working in this school.

For all scales, higher scores reflect greater levels of the construct being measured.

#### Teacher Self-Efficacy

Teacher self-efficacy for autism was assessed using a four-item scale adapted from a five-item measure that the present authors previously co-produced for evaluation of *Autism in Schools and Settings* with a local County Council, a national autism charity and stakeholder representatives, drawing on Bandura’s (1997) self-efficacy framework and existing measures of teacher self-efficacy for inclusive practice. The scale was designed to assess staff members’ perceived understanding of autistic pupils’ strengths and needs, confidence in supporting autistic pupils in their role, knowledge of recommended strategies, access to advice and resources, and the perceived importance of supporting autistic pupils in their setting. For the current study, we retained the four items indexing personal capability: *I have a high level of understanding of the strengths and needs that autistic young people may have; I feel very confident supporting autistic young people in my role; I know lots of recommended ways to support autistic young people; I can easily access advice and resources for supporting autistic young people*. Items were rated on a 5-point scale (1 = *strongly disagree*; 5 = *strongly agree*). Total score ranged from 4 to 20. This scale had a Cronbach’s alpha coefficient of .91, indicating excellent internal consistency.

#### Intentions to Implement Inclusive Practice (Completed by all participants except support staff)

The Intention to Teach in Inclusive Classrooms Scale (ITICS) (Sharma & Jacobs, 2016) was adapted for this survey specifically to refer to autistic pupils, to more precisely assess teachers’ motivation and planned behaviour in this context. This decision was grounded in Ajzen’s Theory of Planned Behaviour, which highlights intention as a key predictor of actual practice. The adapted version was made up of 5 items measuring teachers’ likelihood of implementing inclusive practices, such as *include autistic students with greater support needs in a range of activities in your class* and *adapt the physical environment to decrease sensory overload for autistic pupils*. Items were rated on a 5-point scale (1 = *strongly disagree*; 5 = *strongly agree*). Total score ranged from 5 to 25. The scale had an acceptable internal consistency, with a Cronbach’s alpha coefficient of .70.

#### Neurodiversity-Affirming Attitudes

A 14-item scale, with seven items drawn from the Autism Inclusion Questionnaire (AIQ; Segall, 2011, adapted from Segall and Campbell (2007)) and seven items adapted from (a) Educators’ Beliefs about Diversity in Personal and Professional Contexts scale – selected and adapted from Pohan and Aguilar (2001) – to reflect neurodiversity rather than the other kinds of diversity; and (b) Learning to Teach for Social Justice-Beliefs (LTSJ-B) scale (Enterline et al., 2008) – adapted for neurodiversity. Items were rated on a 5-point scale (1 = *strongly disagree*; 5 = *strongly agree*). We aggregated the 14 items into a single composite of neurodiversity-affirming attitudes because all items were selected to index one construct; internal consistency was acceptable (α = .78), supporting use of a total score ranging from 14 to 70.

#### Perceptions of Inclusive School Environment

Seventeen items were selected from the Parents’ Perceptions of Inclusive School Quality (PISQ), specifically the physical environment and psychological environment subscales (Zhao et al., 2021). The scale was adapted for staff and content was reviewed by school leaders/SENCos to ensure relevance. Because school climate/inclusion are multi-informant constructs, staff are appropriate raters of the same underlying school-level features. The scale measures views on the inclusiveness and quality of schools. Its physical environment subscale assesses the accessibility and suitability of school facilities, whereas the psychological environment subscale evaluates the emotional safety, inclusiveness, and social climate of the school. Items were rated on a 5-point scale (1 = *strongly disagree*; 5 = *strongly agree*). Total score ranged from 17 to 85. The scale has excellent reliability with a Cronbach’s alpha of .92 for this dataset.

#### Reasonable Adjustments and Perceived Challenges

Three questions with open-text responses asked participants for (1) examples of reasonable adjustments that could be used to support autistic pupils’ needs and how likely they would be to use them, (2) perceived difficulties in supporting autistic pupils in their role, and (3) *for participants who reported receiving autism-specific training from an external provider –* descriptions of any changes they had made to their practice following this training, to explore the ways in which professional development may influence day-to-day support for autistic pupils.

### Data Analysis

Hierarchical multiple regression was used to examine the incremental contribution of theoretically distinct predictors of teacher self-efficacy and intentions to implement inclusive practice. Demographic and background variables (gender, role, years worked in the school, and personal connection to autism) were entered at *Step 1* as control variables. *Step 2* included autism-specific training and direct experience with autistic pupils to represent knowledge- and exposure-based factors. *Step 3* included neurodiversity-affirming attitudes and perceptions of the inclusive school environment, reflecting more proximal attitudinal and contextual influences on inclusive practice. All analyses were conducted in SPSS v.29.

Responses to the three open-text questions – requesting examples of reasonable adjustments, perceived difficulties in supporting autistic pupils and changes to practice following specialist training – were analysed using thematic analysis (Braun & Clarke, 2006, 2021). The analysis followed the six-phase approach: familiarisation with the data, generation of initial codes, searching for themes, reviewing themes, defining and naming themes, and producing the report. An inductive, semantic approach was adopted, focusing on the explicit content of participants’ responses rather than underlying assumptions. All responses were read and coded manually, with recurring patterns identified and grouped into overarching themes to capture key aspects of the data relevant to the research aims. Thematic analysis was led by the first author, who generated the initial codebook. To enhance credibility, a second researcher independently reviewed and recoded a subset of responses using this framework; discrepancies and ambiguities were discussed and the coding structure refined accordingly.

### Positionality Statement

This study was conducted within a commitment to participatory, reflexive, and neurodiversity-affirming autism research, with a specific focus on understanding factors that shape teachers’ self-efficacy and inclusive practice in mainstream schools. The first author is a parent of two autistic young people, bringing lived experience of navigating educational systems, teacher–family relationships, and the variability of inclusive practice across school contexts. The second author is AuDHD, contributing an insider perspective on neurodivergent schooling experiences and the ways in which teacher attitudes, expectations, and environmental fit can meaningfully influence participation and well-being.

These positions informed our interest in examining teachers’ confidence, training, and intentions to implement inclusive practice, and shaped our emphasis on neurodiversity-affirming attitudes and the perceived inclusivity of school environments as potentially proximal influences on teachers’ practice.

We recognise that our identities and experiences may shape the research questions posed, the constructs prioritised, and the interpretations drawn. To support reflexivity and accountability, we worked iteratively with colleagues from the United Kingdom’s National Autistic Society (NAS) throughout study design, measure review, and interpretation of findings, and incorporated mixed methods to ensure that participants’ accounts of training, challenges, and practice could contextualise and nuance quantitative results. We acknowledge that we are not positioned to represent the full diversity of educational experiences across the UK. Accordingly, the findings are situated specifically within mainstream secondary school contexts in Southeast England, within the English education system, and offer insight into ongoing efforts to strengthen teacher capacity for inclusive practice.

### Participatory Methods

To strengthen accountability and centre autistic perspectives, we worked iteratively with the NAS, a leading U.K.-based autism charity, throughout study design and interpretation of findings. During the design phase, NAS researchers provided feedback on the selection of measures. While analytical decisions and interpretation of the final dataset remained the responsibility of the academic research team, consultation with the NAS offered valued feedback to contextualise results, reflect on the limitations within our data, and strengthen interpretation.

## Results

### Data Screening

Missing data ranged from 0% to 25% across items within individual scales. Missing value analysis confirmed that data were missing completely at random. If 25% of items or less were missing in a single scale, the mean of the remaining scores was imputed. Otherwise, pairwise deletion of missing data was implemented. Preliminary analyses were conducted to ensure no violation of the assumptions of normality, linearity, multicollinearity, and homoscedasticity. Alpha was set at .05.

### Descriptive Statistics

Table 1 provides the means and standard deviations of study variables.

**Table 1.** Descriptive Statistics for Study Variables (*N* = 126).

Variable	*M*	*SD*
Teacher self-efficacy	3.78	0.82
Intentions to implement inclusive practice	3.98	0.62
Neurodiversity-affirming attitudes	4.16	0.35
Perception of inclusive school environment	3.95	0.61

### Correlations

Bivariate correlations revealed several meaningful relationships between the study variables, with small to moderate effect sizes (see Table 2). Teacher self-efficacy was positively associated with intentions to implement inclusive practice, specialist autism training, being a TA, neurodiversity-affirmative attitudes, and higher perceived quality of inclusive school environment. Intentions to implement inclusive practices were also positively related to inclusive attitudes, specialist training, personal connection to autism, and being a TA.

**Table 2.** Bivariate Correlations Among Study Variables.

Variable	2.	3.	4.	5.	6.	7.	8.	9.	10.	11.	12.
1. Teacher Self-Efficacy	.18*	.09	−.07	.32**	−.06	−.08	.03	.48***	.16	.26**	.24*
2. Intentions to implement inclusive practice		.14	.11	.23*	−	−.13	.25**	.39***	−.15	.41***	−.10
3. Gender			−.07	.16	.11	−.04	−.11	.24*	−.04	.01	−.09
4. Leadership vs. CT				−.20*	−.17	.30**	.09	.21*	.23*	.18	.26**
5. TA/LSA vs. CT					−.18*	−.21*	.18	.45***	−.42***	−.03	−.22*
6. Support Staff vs. CT						.08	.06	−.03	.05	−.04	.03
7. Years at school							−.15	.13	.39***	.04	.17
8. Personal connection								.11	−.25*	.003	−.19*
9. Specialist training									.04	.19*	−.09
10. Time working with autistic pupils										−.02	.26**
11. Neurodiversity-affirmating attitudes											.16
12. Quality of inclusive environment											

*Note.* CT = classroom teacher; TA = teaching assistant; LSA = learning support assistant; ND = neurodiversity.

* *p* < .05. **p < .01. ***p < .001.

Other noteworthy findings indicate role-related differences in perceptions of the inclusive environment, with higher ratings among leaders and lower ratings among TAs, and specialist training being associated with more neurodiversity-affirmative attitudes.

### Predictors of Teacher Self-Efficacy

The combined set of predictor variables explained 42.1% of the variance in self-efficacy, *F*(10, 76) = 7.26, *p* < .001. After controlling for gender, role, years working as a teacher, and personal connection to autism, five variables emerged as unique predictors of teacher self-efficacy in supporting autistic pupils. Autism-specific training was the strongest predictor (β = .43, *p* < .001), accounting for 10.8% of the unique variance. Perceived quality of the inclusive environment was the second strongest predictor (β = .34, *p* < .001; 9.4% unique variance), followed by length of time working with autistic pupils (β = .30, *p* = .004; 5.8% unique variance). Having a classroom teacher role, compared with a leadership role, was also a significant predictor (β = –.72, *p* = .008; 5.0% unique variance), with the negative beta reflecting the coding of leadership relative to classroom teachers, indicating that classroom teachers reported higher self-efficacy than leaders. Finally, neurodiversity-affirming attitudes predicted higher self-efficacy (β = .19, *p* = .03; 3.1% unique variance).

### Predictors of Intentions to Implement Inclusive Practice (Completed by all participants except support staff)

The combined set of predictor variables explained 27.5% of the variance in intentions to implement inclusive practice, *F*(9, 84) = 4.91, *p* < .001. After controlling for gender, role, years working at the school, and personal connection to autism, two variables emerged as unique predictors of inclusive practice in supporting autistic pupils. Neurodiversity-affirming attitudes were the strongest predictor (β = .37, *p* < .001), accounting for 12.7% of the unique variance. Autism-specific training also predicted inclusive practice (β = .25, *p* = .02; 4.2% unique variance). Personal connection to autism, length of time working with autistic pupils, and quality of the inclusive environment were not significant predictors.

### Qualitative Findings

#### 1. Reasonable Adjustments to Support Autistic Pupils

Participants provided examples of reasonable adjustments. Analysis identified three overarching themes:

Facilitating Clear Communication and Structured Learning *(56% of examples; higher proportion from TA/Learning Support Assistants (LSAs)).* Participants most frequently described adjustments focused on clarity and structure, including the use of short, chunked instructions supported by visual or written materials. Predictability was emphasised through consistent routines and pre-warning pupils about changes to lessons or the school day to reduce anxiety and support engagement.
Creating Inclusive Physical and Sensory Environments *(29% of examples; higher proportion from those with a personal connection to autism, those in a leadership and TA/LSA roles).* Staff described adapting physical environments to reduce sensory overload, including managing classroom noise and lighting, adjusting seating arrangements, and providing quiet spaces for regulation or breaks. Flexibility in uniform policies was also highlighted as a way of accommodating sensory sensitivities.
Supporting Emotional Regulation and Social Engagement *(15% of examples; higher proportion from those with a personal connection to autism and those who received specialist autism training).* Participants reported strategies such as social stories, structured breaks, and access to low-stimulation spaces. Emphasis was placed on recognising individual triggers, embedding emotional support within daily routines, and fostering inclusive opportunities for peer interaction.

Participants rated their likelihood of using each adjustment on a scale from 1 (*very unlikely*) to 5 (*very likely*). Figure 1 presents category frequencies and mean likelihood ratings.

**Figure 1.**
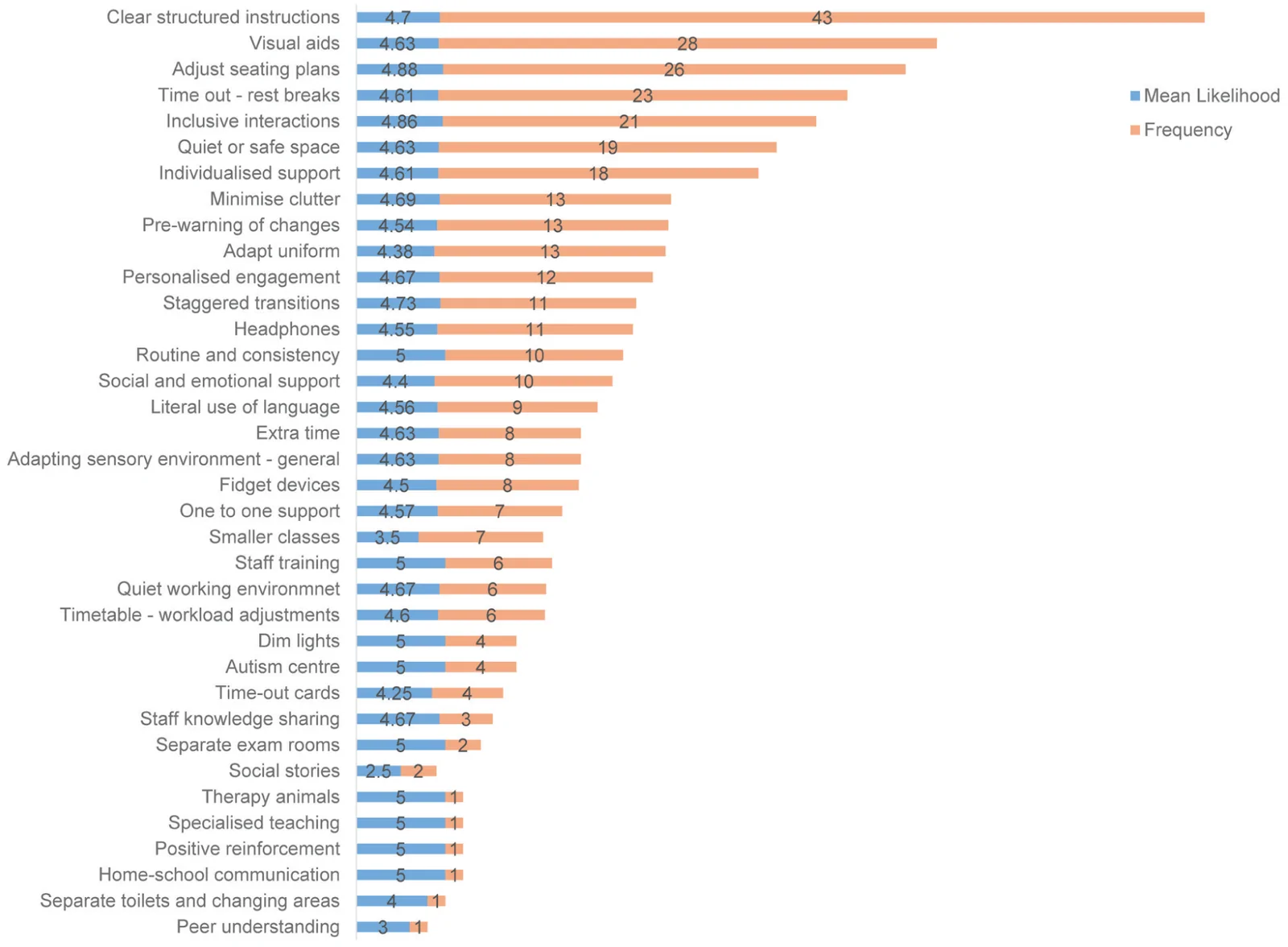
Frequency of reported reasonable adjustment categories and mean likelihood ratings for their use.

#### 2. Challenges in Supporting Autistic pupils

Four overarching themes were identified reflecting challenges in supporting autistic pupils:

##### Individualisation and Understanding of Needs

Participants highlighted difficulties tailoring support to diverse needs. As one participant noted, “Every autistic child is different, so what may work for one may not work for another. Getting to know your students is paramount” (Leadership). Others described uncertainty “between making excuses for undesirable behaviours and making reasonable adjustments” (Classroom Teacher). Support staff raised concerns about limited curriculum adaptation, with one TA stating that “teachers do not change the subject material for children with additional needs.” Limited access to background information also constrained support, as one teacher explained, “Not having the full picture of their situation – unsure when and how best to support them.” These challenges were sometimes linked to training needs: “I also lack training of techniques for students with greater struggles” (Leadership).

##### Time and Resource Constraints

Time pressure, class size, and staffing limitations were reported barriers. One teacher described the challenge of supporting “30 students in a class, 3 autistic students – you teach to the masses or you don’t teach” (Classroom Teacher). Support staff highlighted the impact of staffing instability, where “staff shortages can mean that timetables are shifting/inconsistent” (TA). Although one leader stated that “time is always going to be an issue, but reasonable adjustments do not take much time,” TAs described limited time, skills, and organisational support as restricting implementation. One participant referred to “leadership attitudes/education not allowing adjustments. Or school rules preventing changes being made,” whereas another identified the main difficulty as “not having the time and skills to be able to support more. So much more could be done to provide a more supportive role to the children.”

##### Classroom Environment and Dynamics

Managing diverse needs in busy classrooms was described as challenging. Teachers referred to “juggling the needs of different pupils e.g. those who have noisy stims or ADHD pupils with fidget toys with the sensory needs of others who find extraneous sound distracting can be difficult” (Classroom Teacher), alongside “managing the behaviour of other students and lacking a clear sanctioning pathway for students with autism” (Support Staff). Certain environments posed particular challenges, such as “the Food room is a very busy, noisy, practical environment with up to 24 students in each class. If a student is unable to even be in that room, there is little we can do” (Support Staff). Leaders noted that a “mainstream” environment “is not designed to best support ASD children – [school] is an especially vibrant and busy place, which sometimes makes the challenges more difficult.” A PE teacher similarly described difficulties “ensuring a stable, regular environment is upheld as we are regularly changing facilities we are using.”

##### Systemic and Institutional Barriers

Participants reported gaps in training and inconsistent institutional support. Some described receiving “no training based around more up-to-date neurodivergent-affirming methods or interventions” (TA), whereas others cited a “lack of communication around specific students’ needs and lack of consistency between departments in terms of strategies” (TA). System-level barriers were also identified. One leader reflected that

the current set up of SEND provision in the UK is difficult to navigate for children with significant levels of need. This is either because their needs are not severe enough to make them a fit for special or because they are a good fit for special, but there are not enough special places. There seems to be a lot of categorisation of children and fitting square pegs into round holes. With more flex, the system would likely be more effective.

A lack of senior leadership support was also described, with one classroom teacher reporting “the lack of understanding and real support from SLT, not the purported understanding/support, and the drive for academic results which doesn’t always allow for the individuals who learn differently.”

#### 3. Changes in Practice Following Training

Of the 38 participants (30.2%) who had received autism-specific training from external providers, most reported changes to practice including shifts in communication, environmental adaptation, individualised approaches, and reflective professional practice.

##### Enhanced Communication Strategies

Participants described using clearer, more direct language, avoiding idiomatic expressions, and breaking instructions into smaller steps supported by written or visual prompts. One leader described “giving clear instructions with no idioms, repeating if needed and also writing down instructions or giving a writing frame or notes, as needed . . . [and] signposting at the start of the lesson and explaining the purpose.” A classroom teacher similarly noted “limiting the number of instructions given, checking in on mental welfare subtly but more frequently, allowing more time for tasks, [and] bonding tasks, including home learning.” Support staff also reported using “clearer, unambiguous, concise instructions” and giving pupils time to process by “walk[ing] away and return[ing] when the student is ready.”

##### Increased Sensory Awareness and Adaptation

Training increased awareness of sensory processing differences. Participants described reducing noise, adjusting lighting, decluttering spaces, and providing quieter areas. A TA reported, “I never challenge any of my students on uniform as I know this is a source of distress for many neurodivergent students.” One participant described a broader shift:

I was more aware of potential sensory needs, only switching the lights on if needed, warning before doing so. Keeping the area quiet and respectful, challenging any unkindness or intolerance even if not directed. Awareness of different needs when going through stressful times. (Support Staff)

##### Individualised and Flexible Approaches

Training encouraged more individualised responses. This included adapting seating arrangements and adjusting routines to meet individual needs. One leader described becoming “more sensitive to their individual needs, picking up on any anxieties or difficulties and checking in on them more but not intrusively.” Another reflected,

I have also worked closely with a sixth form student this academic year and realised that ways in which I have tried to support them haven’t always been as supportive to them as I intended, i.e., flexibility. *(Leadership)*

A TA noted a similar change in perspective: “I am more mindful of students experiencing meltdowns vs shutdowns and how to alter my language and response depending [on] which situation is occurring.”

##### Advocacy and Reflective Practice

Several participants reported feeling more confident in advocating for autistic pupils following training. This included raising awareness among colleagues, influencing policy discussions, and ensuring autistic voices were heard in decision-making processes. One support staff member described,

Doing the NAS Teen Mental Health Course introduced me to new terminology. It seemed to simplify the knowledge I had already which has made it possible for me to explain to other colleagues what may be going on for those young people. The training has made me feel I can advocate more strongly for the students I see. It has given me the language to describe what I knew already from experience. *(Support Staff)*

Leaders also reported greater reflection, with one stating that training had “reminded me that each child experiences things differently; that what to me is a small event might be huge for the child . . . [and that] social cues will not always be read in the same way.” A classroom teacher similarly described “being more reflective of my interactions and what effect they have on other individuals, both children and adults; making adaptations should there be overwhelm; [and] listening to individual voices more.”

## Discussion

The present study set out to address a well-documented problem in mainstream secondary education: autistic pupils’ needs are frequently unmet, despite strong policy commitments to inclusive education. Existing evidence indicates that teachers’ preparedness, attitudes, and perceptions of their school environment all shape their ability to deliver inclusive practice (e.g., Collie et al., 2012; Forlin et al., 2014; Humphrey & Lewis, 2008; Yada et al., 2022), yet most prior studies have examined these influences in isolation or with a limited set of predictors. This leaves gaps in understanding the relative contribution of different factors when considered simultaneously.

Our study responded to these gaps by incorporating a broad range of theoretically and empirically grounded predictors – including autism-specific training, inclusive school environment quality, years of experience with autistic pupils, neurodiversity-affirming attitudes, personal connection to autism, and staff role – into a single model predicting two key outcomes: teachers’ self-efficacy for autism and intentions to implement inclusive practices. By adopting this design, the study not only quantified the influence of individual predictors but also compared their relative strength within the same analytical framework. This integrated approach advances the field’s understanding of how professional skills, values, and contextual factors combine to influence teachers’ confidence and motivation to include autistic pupils.

### Predictors of Teachers’ Self-Efficacy for Autism

The regression analysis revealed that autism-specific training was the strongest unique predictor of teachers’ self-efficacy, explaining the largest proportion of unique variance (10.8%). This finding contrasts with earlier work (Hamilton & Cook, 2026), in which training did not emerge as a significant predictor of self-efficacy. In that study, however, training was captured using a binary indicator that did not account for substantial variation in its duration, provision or specificity. In the current study, the stronger predictive value of autism-specific training may therefore reflect a more focused conceptualisation of training. This finding aligns closely with prior studies showing that targeted professional development improves both confidence and competence in inclusive education (Alassaf, 2025; Boyle et al., 2013; Forlin et al., 2014). Importantly, our qualitative data added depth to this relationship, illustrating how specialist autism training translated into concrete changes in practice: clearer and more explicit communication, heightened sensory awareness, individualised approaches, and reflective practice. These reported shifts resonate with earlier research indicating that professional development not only enhances knowledge but also prompts more adaptive, student-centred pedagogical choices (Nolan & Hannah, 2019; Love et al., 2019).

The second strongest predictor of teachers’ self-efficacy was perceived quality of the inclusive school environment (unique contribution 9.4%). This echoes evidence that an inclusive organisational climate – characterised by supportive leadership, collegial trust, and resource availability – bolsters teachers’ capacity to address diverse needs (Collie et al., 2012; Hoy & Woolfolk, 1993; Pas et al., 2012). Our findings suggest that such environmental affordances may complement individual skill-building, enabling teachers to build their confidence in meeting the needs of autistic pupils.

Years of direct experience with autistic pupils emerged as the third predictor (unique contribution 5.8%), consistent with the notion of mastery experiences as an influential source of efficacy beliefs (Bandura, 1997). Teachers with more than 5 years’ experience may have encountered a broader range of pupil needs and developed a repertoire of strategies to address them, in line with studies showing that meaningful exposure to autistic pupils can improve confidence and reduce anxiety (Bešić et al., 2017). However, as noted in the introduction, the literature on this relationship is mixed (Ruble et al., 2011), suggesting that quality and diversity of experience may matter as much as duration.

Staff role was also a significant predictor, with classroom teachers reporting higher self-efficacy than those in leadership roles. This indicates that direct, regular classroom engagement may enhance perceived competence in ways that strategic oversight does not. This pattern mirrors earlier findings that proximity to day-to-day teaching tasks contributes to efficacy beliefs (Symes & Humphrey, 2011). One plausible explanation is that our self-efficacy scale focused on perceived understanding of autistic pupils’ strengths and needs, confidence in supporting them in one’s own role, knowledge of recommended strategies, and ease of accessing advice and resources – domains that are likely to be shaped by regular, hands-on work with autistic pupils and day-to-day problem-solving, which are typically more central to classroom teachers’ practice than to senior leaders’. From a social-cognitive perspective, efficacy beliefs are shaped largely by repeated mastery experiences in the relevant domain (Sharma & George, 2016), so staff who teach autistic pupils regularly may accumulate more successful experiences to draw upon when judging their capabilities. Conversely, leaders typically have less frequent direct contact with autistic pupils and a more strategic remit, which may heighten awareness of structural and resource constraints on inclusion; this broader perspective could temper their personal efficacy ratings even when they are strongly committed to inclusive education.

Finally, neurodiversity-affirming attitudes, while contributing a smaller proportion of unique variance (3.1%), were nonetheless a significant predictor. This suggests that valuing neurological difference, framing autism through a strengths-based lens, and resisting deficit-focused narratives may positively shape teachers’ sense of capability. Although empirical research on neurodiversity-affirming attitudes remains limited, our findings align with broader evidence linking positive inclusion attitudes to higher self-efficacy (Avramidis & Norwich, 2002; Yada et al., 2022).

### Predictors of Intentions to Implement Inclusive Practices

For intentions to implement inclusive practices, the predictor hierarchy shifted. Neurodiversity-affirming attitudes were the strongest unique predictor (12.7%), followed by autism-specific training (4.2%). This pattern underscores the motivational role of values and beliefs in shaping planned behaviour, consistent with Ajzen’s Theory of Planned Behaviour, which posits that attitudes directly influence intention. Teachers who endorse neurodiversity principles may be more intrinsically motivated to adapt practice, even in the absence of structural or environmental supports.

The association between training and intentions mirrors prior work demonstrating that professional development not only builds skills but also fosters commitment to inclusion (Forlin et al., 2014). Interestingly, inclusive school environment quality, experience with autistic pupils, and personal connection to autism did not predict intentions when other variables were controlled. This suggests that while environmental and experiential factors may boost self-efficacy, the decision to implement inclusive strategies is more strongly linked to intrinsic values and targeted skill acquisition.

### Qualitative Insights and Practical Implications

The qualitative findings show how staff operationalise inclusive practice day-to-day. Staff described a wide range of reasonable adjustments, which broadly fell into three overlapping areas: clear communication and structured learning, creating inclusive physical and sensory environments, and supporting emotional regulation and social engagement. These strategies closely align with prior research showing that predictability, environmental adaptation, and emotional regulation support enhance autistic pupils’ participation and well-being (Boddy & Cook, 2026; Goodall, 2018; Humphrey & Lewis, 2008; Saggers et al., 2011). That TAs often emphasised detailed, one-to-one adjustments echoes findings that staff in these roles frequently deliver the most individualised support (Symes & Humphrey, 2012; Webster et al., 2013), whereas the greater focus on emotional regulation among staff with personal connections to autism reflects literature linking lived experience with heightened empathy and responsiveness (MacLeod et al., 2017).

Participants also identified substantial challenges to implementing these strategies effectively. A major theme was the difficulty of individualising support within the constraints of busy classrooms, where diverse needs – including conflicting sensory preferences – must be balanced. Limited resources, large class sizes, and staffing shortages were frequently cited, reflecting systemic barriers noted in earlier studies (Avramidis et al., 2000; Florian & Pantic, 2017). Some participants described gaps in communication and consistency between departments, and the absence of whole-school, neurodiversity-affirming approaches, which echoes findings that without coordinated institutional support, inclusion often relies on individual goodwill and initiative (Hosford & O’Sullivan, 2016; Weisel & Dror, 2006). Practical subject areas, such as food technology and PE, were noted as especially challenging due to sensory load or environmental instability. Together, these challenges highlight that while individual training and attitudes are vital, they must be matched by organisational alignment, resource allocation, and leadership support to enable sustained, high-quality inclusion for autistic pupils.

### Limitations

Several limitations should be acknowledged. First, the cross-sectional design precludes causal inference; while training and attitudes were associated with higher self-efficacy and intentions, it is possible that more confident teachers are also more likely to seek training or hold affirming attitudes. Second, self-report measures may be subject to social desirability bias, particularly for attitudes and intentions towards inclusion. Third, while the sample size met the a priori power requirement, it was drawn from a specific geographic region and limited to secondary schools, which may constrain generalisability to other contexts. Fourth, five of the six participating schools had SRCs for autistic pupils. This may limit the generalisability of the findings to schools without such provision, as staff in SRC-equipped schools may have greater access to specialist expertise and resources. (Nonetheless, variability in experiences, attitudes, and perceptions of the school environment was still evident across the sample, suggesting the findings remain informative beyond SRC contexts.) Finally, the geographical scope of the evidence base we draw on is dominated by studies conducted in high-income “Global North” systems such as the United Kingdom and Ireland, Western Europe, North America, Australia/New Zealand, Finland, and Japan, with a smaller number of studies from upper-middle-income contexts including Hong Kong, Singapore, Turkey, Saudi Arabia, India, China, and Israel. Research from low-income and more resource-constrained Global South settings remains scarce, which means current knowledge may not fully capture the realities of inclusion in these contexts.

## Conclusion

This study offers one of the first integrated examinations of multiple theoretically relevant predictors of teachers’ self-efficacy for autism and intentions to implement inclusive practice within U.K. mainstream secondary schools. Autism-specific training and neurodiversity-affirming attitudes emerged as central drivers, with environmental and experiential factors further bolstering self-efficacy. The findings highlight the need for professional development that not only equips teachers with practical strategies but also fosters neurodiversity-affirming values. Equally, they point to the importance of cultivating inclusive school climates that enable teachers to act on their intentions. Addressing systemic barriers – from resource limitations to inconsistent institutional support – will be critical in translating these individual and organisational strengths into consistent, high-quality support for autistic pupils.

## Data Availability

The data that support the findings of this study are not publicly available. Ethical approval and informed consent for this project were obtained on the basis that participants’ data would be used only by the research team and would not be shared beyond it; therefore, the anonymised dataset cannot be deposited in an open repository without breaching the approved protocol and consent conditions.
